# Alabama State Laws Regulating the Practice of Medicine

**Published:** 1881-02

**Authors:** 


ALABAMA STATE LAWS REGULATING THE PRACTICE OF MEDICINE.—We would respectfully call attention to the letter of "Subscriber," in this issue, upon the above subject. Alabama has done nobly and set an example that should be at once followed by all our legislatures. The people elect men to legislate and make laws for them at their state capitols, and generally without knowing in advance what they are going to enact. But if they could be made aware of the vital importance to themselves, and their posterity, of allowing no one to assume the duties and functions of a physician, who is not thoroughly qualified for the office, they would, as with one voice, urge their representatives to the passage of laws prohibiting all others from practicing medicine, or attempting to do so, under the heaviest penalties.

